# Mesenchymal Stem/Stromal Cells in Skeletal Muscle Are Pro-Angiogenic, and the Effect Is Potentiated by Erythropoietin

**DOI:** 10.3390/pharmaceutics15041049

**Published:** 2023-03-24

**Authors:** Yoshitaka Iso, Sayaka Usui, Hiroshi Suzuki

**Affiliations:** Division of Cardiology, Showa University Fujigaoka Hospital, 1-30 Fujigaoka, Yokohama City 227-8501, Kanagawa, Japan

**Keywords:** mesenchymal stem/stromal cells, skeletal muscle, angiogenesis

## Abstract

The aim of this study was to investigate the angiogenic potential of skeletal muscle mesenchymal stem/stromal cells (mMSCs). Platelet-derived growth factor receptor (PDGFR)-α positive mMSCs secreted vascular endothelial growth factor (VEGF) and hepatocyte growth factor when cultured in an ELISA assay. The mMSC-medium significantly induced endothelial tube formation in an in vitro angiogenesis assay. The mMSC implantation promoted capillary growth in rat limb ischemia models. Upon identifying the erythropoietin receptor (Epo-R) in the mMSCs, we examined how Epo affected the cells. Epo stimulation enhanced the phosphorylation of Akt and STAT3 in the mMSCs and significantly promoted cellular proliferation. Next, Epo was directly administered into the rats’ ischemic hindlimb muscles. PDGFR-α positive mMSCs in the interstitial area of muscles expressed VEGF and proliferating cell markers. The proliferating cell index was significantly higher in the ischemic limbs of Epo-treated rats than in untreated controls. Investigations by laser Doppler perfusion imaging and immunohistochemistry demonstrated significantly improved perfusion recovery and capillary growth in the Epo-treated groups versus the control groups. Taken together, the results of this study demonstrated that mMSCs possessed a pro-angiogenic property, were activated by Epo, and potentially contributed to capillary growth in skeletal muscle after ischemic injury.

## 1. Introduction

Chronic critical limb ischemia is the most severe manifestation of peripheral artery disease (PAD) [1]. Therapeutic angiogenesis with bone-marrow-derived and adipose-derived cells has been developed as a less invasive intervention for patients severely compromised by critical limb ischemia in the last two decades [2,3,4]. This approach aims at improving perfusion to the ischemic vascular beds by inducing the formation of new blood vessels from preexisting ones. The cell-based therapies are vital for increasing blood flow to ischemic regions in no-option patients with severe limb ischemia [5].

Adult bone marrow contains not only hematopoietic progenitors but also non-hematopoietic progenitor subsets commonly referred to as mesenchymal stem/progenitor cells or multipotent stromal cells (MSCs). Bone marrow MSCs (BMMSCs) possess attractive properties for use in safe and effective cardiovascular cell therapies and can be easily isolated from bone marrow and expanded in culture systems [6]. BMMSCs are capable of secreting pro-angiogenic and cytoprotective factors, such as vascular endothelial growth factor (VEGF) and fibroblast growth factor (FGF)-2, as well as proteins that modulate endothelial cell migration [7,8,9,10]. Our group previously examined a BMMSC culture system to confirm the angiogenic and endothelial protective effects of the expressed cell factors [7,9]. We found that co-culturing with human BMMSCs induced prominent capillary network formation in an in vitro angiogenesis assay [9].

A growing number of reports have identified MSCs in bone marrow as well as various other tissues [11,12,13,14]. While the properties of MSCs are well understood in the bone marrow and adipose tissue, less is known about them in other parts of the body. The different MSCs derived from skeletal muscle and various other tissues of the body may share reparative abilities as tissue-repairing cells [15]. Human and rat MSCs isolated from skeletal muscle (mMSCs) have a high proliferative capacity, self-renewal ability, and ability to differentiate into osteoblasts, adipocytes, and chondrocytes, as well as BMMSCs [11,12]. Uezumi et al. detected platelet-derived growth factor receptor (PDGFR)-α in mMSCs of mouse muscle interstitium [16]. The mMSCs promote the myogenic differentiation of co-cultured satellite cells and regulate the maintenance of muscle fibers [17,18]. Our previous study indicated that the interaction between the mMSCs and FGF-23 may play a key role in the impaired muscle reparative mechanisms of chronic kidney disease [19].

Owing to the nature of MSCs, mMSCs may also contribute to inducing angiogenesis and repairing injured tissue in an ischemic muscle through the secretion of a repertoire of cytokines and growth factors. However, the angiogenic properties of mMSCs have not been previously investigated. Thus, in this study, we investigated the pro-angiogenic potential of mMSCs. Cell implantation is a useful therapy, but activation of endogenous reparative mechanisms appears less invasive and more attractive. mMSC activation in lower limb muscle could be a novel therapeutic strategy for PAD, including critical limb ischemia. We also investigated whether these cells could be a therapeutic target for the rescue of ischemic hindlimb muscle in rat models. Erythropoietin (Epo) was tested as a candidate agent for stimulating mMSCs because Epo elicited pro-survival potential in BMMSCs in our previous study [10].

## 2. Materials and Methods

### 2.1. Cell Isolation and Culture

Rat mMSCs were kindly provided by Professor Ichiro Sekiya from the Center for Stem Cell and Regenerative Medicine, Tokyo Medical and Dental University. The mMSCs were isolated from rat skeletal muscle as described previously [11]. Briefly, hindlimb skeletal muscles of 12-week-old male Sprague-Dawley rats were excised, digested for 3 h at 37 °C with type II collagenase (0.2%; Sigma, Cream Ridge, NJ, USA), and passed through a 40-μm filter (Becton Dickinson, Franklin Lakes, NJ, USA) to yield single-cell suspensions. Nucleated cells were plated and cultured in complete culture medium (CCM) consisting of αMEM (Life Technologies, Carlsbad, CA, USA), 20% fetal bovine serum (Life Technologies), 100 U/mL penicillin, 100 μg/mL streptomycin, and 2 mM L-glutamine (Life Technologies) and incubated at 37 °C with 5% CO_2_. After 24 h, the nonadherent cells were removed, and the primary adherent cells were cultured and propagated. Rat BMMSCs were also kindly provided by Professor Jeffery L. Spees from the University of Vermont. The BMMSCs were isolated from rat bone marrow as described previously [20]. Briefly, mononuclear cells were purified from rat bone marrow by density centrifugation and re-suspended in CCM. Nucleated cells were plated and then cultured in CCM, like the mMSC culture.

Rat skeletal muscle cells (skMCs) and human umbilical cord vein endothelial cells (HUVECs) were purchased from Cell Applications Inc. (San Diego, CA, USA). Each cell type was cultured under the appropriate conditions. All experiments were performed using cells from the 5th to 8th passage.

For the proliferation assay, mMSCs, skMCs, or HUVECs were plated at a density of 5000 cells/cm^2^ in 20 mL of the appropriate medium in a 150 cm^2^ culture dish. Recombinant erythropoietin was kindly provided by Chugai Pharmaceutical Co., Ltd. (Tokyo, Japan). The cells were cultured in a normoxic humidified incubator (ASTEC, SCA-165DS) with 95% air and 5% CO_2_ at 37 °C and then treated with and without Epo (80 IU/mL) (n = 3 to 6). The mMSCs and skMCs were detached by trypsin and counted after 2 days; the HUVECs were detached and counted after 5 days. The cell count ratio was defined as the ratio between the number of cells with and without Epo treatment.

### 2.2. Preparation of Conditioned Medium

The conditioned medium was prepared as previously described [7,21]. In brief, the cells grown to 70–80% confluence in 150 cm^2^ dishes were washed with PBS (3 times) and incubated for 48 h with 15 mL of fresh alpha-MEM without any serum or supplements (N = 3). The culture supernatant was then collected, filtered, and stored at −80 °C.

### 2.3. ELISA for Angiogenic Cytokines

To compare the secretion of angiogenic factors among mMSC, skMCs, and BMMSCs, the concentrations of VEGF and hepatocyte growth factor (HGF) were determined in the conditioned media for each cell type by enzyme-linked immunoassay kits (Quantikine, R&D Systems) according to the manufacturer’s instructions.

### 2.4. Immunofluorescence

The mMSCs were cultured in the CCM in a Chamber Slide^®^ (Lab-Tak, CO, USA). Immunofluorescence was observed as previously described [10,19]. The cultured cells were rinsed with PBS, fixed in 4% paraformaldehyde for 10 min, incubated with the first antibody at 4 °C overnight, and subjected to secondary staining. The primary antibodies used were the PDGFR-α antibody (Abcam, MA, USA) at a dilution of 1:500 for 60 min at room temperature and the Epo receptor (Epo-R) (Novus Biologicals, CO, USA) at a dilution of 1:1000. The secondary antibodies used were an Alexa Fluor^TM^ 488-labeled IgG (green color, Life Technologies) and an Alexa Fluor^TM^ 594-labeled IgG (red color, Life Technologies) at dilutions of 1:1000. Nuclear staining was performed using DAPI.

The immunofluorescence of the histological sections was also observed, as previously described [10,21]. The primary antibodies were raised against proliferating cell nuclear antigen (PCNA) at a dilution of 1:100 (DAKO, CA, USA), PDGFR-α at a dilution of 1:500 (Abcam), and VEGF at a dilution of 1: 100 (Santa Cruz Biotechnology, CA, USA). The secondary antibodies were treated via the same procedure described above. Slides were mounted in Vectashield with DAPI (Vector Labs, CA, USA).

### 2.5. Western Blot Analysis

Western blotting was performed as previously described [19], cell lysates were subjected to SDS/PAGE (4–12% gradient gel), and proteins were transferred to ImmunoBlot^TM^ PVDF membranes (0.2 μm; Life Technologies). After blocking, the membrane was incubated overnight at 4 °C with primary antibodies against Akt (1:1000 dilution, Cell Signaling, MA, USA), phospho-Akt (1:1000 dilution, Cell Signaling), STAT3 (1:1000 dilution, Cell Signaling), and phospho-STAT3 (1:1000 dilution, Cell Signaling). Next, the membrane was incubated with HRP-conjugated anti-mouse or anti-rabbit secondary antibody (1:2000 dilution, Santa Cruz Biotechnology), washed, and developed using an enhanced chemiluminescence reagent (Santa Cruz Biotechnology).

### 2.6. In Vitro Tube Formation Assay

Tube formation experiments were conducted using an angiogenesis kit (Kurabo, Osaka, Japan) as previously described [9,22]. Briefly, HUVECs and human fibroblasts were admixed, seeded into 24-well plates, and cultured in either a serum-free medium or the conditioned medium from the mMSCs (n = 4 in each group). After 11 days of culture, the HUVECs were fixed with 70% ethanol at 4 °C and immunostained with an anti-human CD31 antibody using BCIP/NBT as a substrate for the secondary antibody. Capillary-like tube formation was assessed by photography under an inverted phase contrast microscope at a ×40 magnification. Five fields were selected per well for digital photography under a microscope (Olympus, Tokyo, Japan). Angiogenesis image analyzer software (Kurabo) was used to quantitatively measure the areas of the endothelial tubule-like structures (EC area). The EC area ratio was defined as the ratio of the area in the mMSC-medium to that in the control.

### 2.7. Rat Hindlimb Ischemia Model

Hindlimb ischemia was induced by ligating the right femoral arteries of 8-week-old male rats under anesthesia as previously described [10,22]. The distal portion of the saphenous artery and all of its side branches were ligated, along with the veins. The left hindlimb was kept intact and used as a non-ischemic limb.

For the cell implantation study, mMSCs (5 × 10^6^ cells, n = 9) or vehicle (control, n = 9) were injected into the ischemic adductor muscle at four sites immediately after the ligation. Tissue samples were obtained from rat ischemic adductor muscles on the 7th postoperative day for an immunohistochemical study.

For the Epo-direct injection study, Epo (5000 IU/kg, Chugai Pharmaceutical Co., Ltd.) or vehicle (n = 11 in each group) was injected into the ischemic adductor muscle immediately and 7 days after the surgery. Blood perfusion was assessed using laser Doppler perfusion imaging (LDPI) (Omega Zone, Muromachi, Tokyo, Japan) immediately after the procedure and on days 3, 7, and 14 thereafter. The blood flow distribution of the limb was then mapped out as a color-coded image directly proportional to the blood flow perfusion. The lower limb area (femur and below-knee portion) was traced for blood flow quantification in the traced images. The LDPI index was used to calculate the blood perfusion ratio of the ischemic and non-ischemic hindlimbs [10,22]. Tissue samples were obtained from rat ischemic adductor muscles 14 days after surgery for immunohistochemistry.

### 2.8. Quantification of Capillary Vessels and Proliferating Cells

Immunohistochemistry for CD31 and Ki67 was performed to detect capillary vessels and proliferating cells in ischemic rat limbs as previously described [10,22]. Five μm sections were deparaffinized and subjected to a 3-step staining procedure using a streptavidin-biotin complex with horseradish peroxidase. Horseradish peroxidase activity was visualized with a diaminobenzidine substrate, and the sections were faintly counterstained with hematoxylin. The primary antibodies were against CD31 (1:100 dilution, DAKO) and Ki67 (1:100 dilution, Abcam).

The number of CD31-positive cells was counted in three randomly selected fields from each tissue section. The capillary number adjusted per muscle fiber was used to compare differences in capillary density. The proliferating cell index was defined as the ratio of Ki67-positive cells to the total number of cells in each perivascular interstitial area in muscle tissues.

### 2.9. Statistical Analysis

All data were expressed as means ± SD. Comparisons of parameters among the three groups were performed using one-way ANOVA followed by the posthoc test. Comparisons of parameters between the two groups were performed using the unpaired Student’s *t-*test. A *p*-value of < 0.05 was considered significant.

## 3. Results

### 3.1. Pro-Angiogenic Potential in Cultured Rat MSCs Extracted from Skeletal Muscle

The mMSCs were isolated from rat skeletal muscle and cultured in the CCM. The cells were previously shown to be positive for CD90 and negative for hematopoietic lineage markers and were also confirmed to be differentiated into adipocytes, osteocytes, and chondrocytes [11].

**Figure 1:**
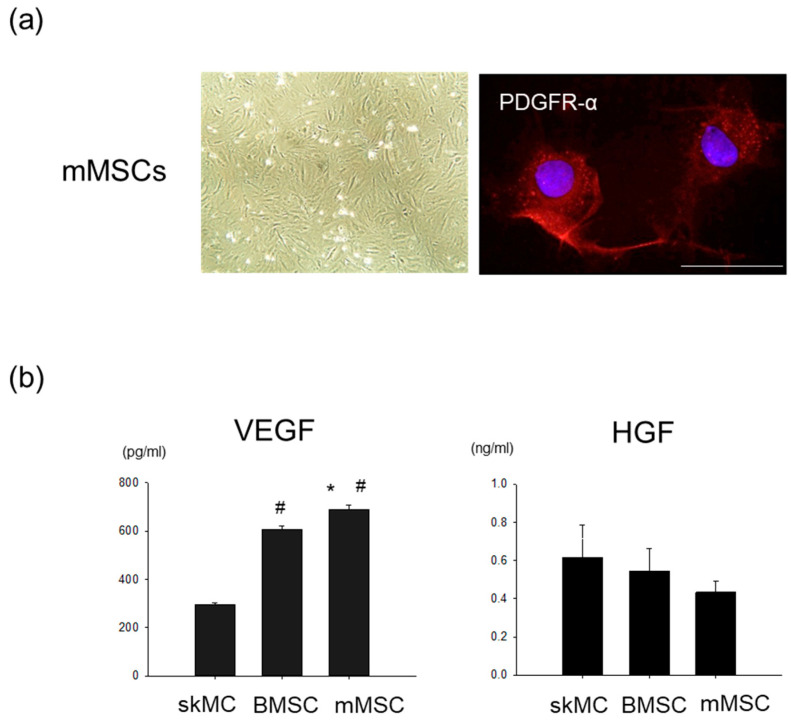
Production of angiogenic cytokines in mMSCs. (**a**) Representative microscopic images of the mMSCs. Left phase contrast image. Original magnification ×40. Right, red fluorescence indicates PDGFR-α. PDGFR-α was ubiquitously expressed in the mMSCs. Nuclei were stained with DAPI (blue). Bar = 50 µm. (**b**) Concentration levels of VEGF and HGF in the conditioned medium. N = 6. * *p* < 0.05 vs. BMMSCs. # *p* < 0.01 vs. skMCs. mMSCs, mesenchymal stem/stromal cells derived from skeletal muscle; PDGFR-α, platelet-derived growth factor receptor-α; DAPI, 4’,6-Diamidino-2-phenylindole; VEGF, vascular endothelial growth factor; HGF, hepatocyte growth factor; BMMSCs, bone marrow mesenchymal stem/stromal cells; skMCs, skeletal muscle cells.

The rat mMSCs exhibited a fibroblast-like morphology and immunopositivity for PDGFR-α (Figure 1a). ELISA was used to determine the VEGF and HGF concentrations in a conditioned medium of the mMSCs, BMMSCs, and skMCs. The VEGF levels were significantly higher in the mMSC-medium than in the BMMSC and skMC mediums, while HGF levels showed no significant differences among the three cell types (Figure 1b).

**Figure 2:**
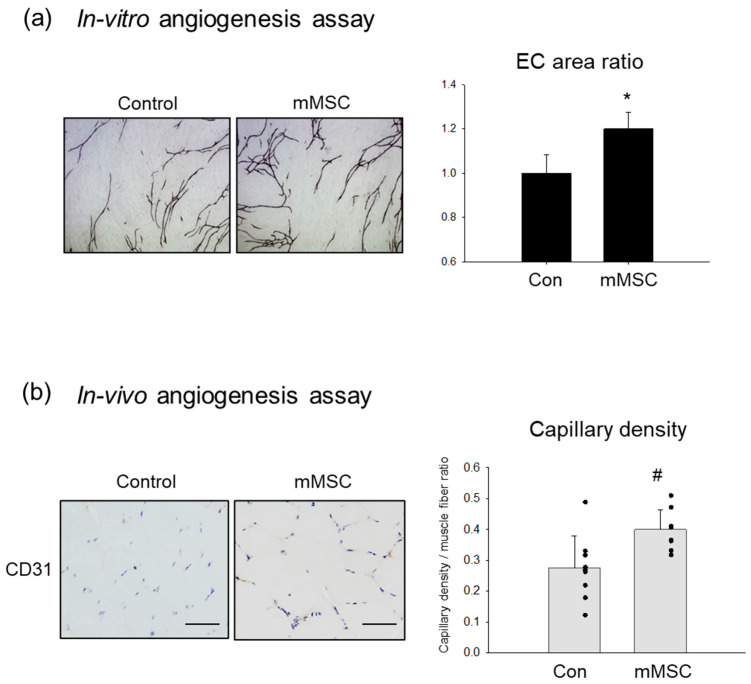
Angiogenic property of mMSCs in vitro and in vivo. (**a**) In vitro angiogenesis assay. Left, images of endothelial network immunostained with CD31 antibody. Original magnification ×40. Right graph, comparison of the areas of the endothelial tubule-like structures (EC area) between the control and conditioned medium from mMSCs. N = 4. * *p* < 0.05. (**b**) In vivo angiogenesis assay in a cell implantation model. Left, immunohistochemistry was performed using CD31 antibody to evaluate the capillaries in skeletal muscle on day 7. Right graph, comparison of the capillary density between the ischemic limbs treated with and without the mMSC implantation. N = 9. # *p* < 0.01. Bar = 50 µm.

An in vitro angiogenesis assay was performed to examine the effect of factors secreted from the mMSCs. The colored areas of HUVECs immunostained with an anti-CD31 antibody were quantified as capillary growth. The conditioned medium from the mMSCs promoted capillary network formation (Figure 2a). The quantitative capillary area was significantly larger in the mMSC-medium than in the controls. To assess the angiogenic property in vivo, cell implantation was performed. Five million mMSCs were locally injected into the ischemic limbs of rats. After 7 days, mMSC implantation significantly increased capillary density in the ischemic limbs compared with that in the controls (Figure 2b).

The mMSCs were pro-angiogenic and promoted capillary growth in vitro and in vivo. This is potentially because of a paracrine action of the secreted factors.

### 3.2. Effects of Epo on mMSCs In Vitro

**Figure 3:**
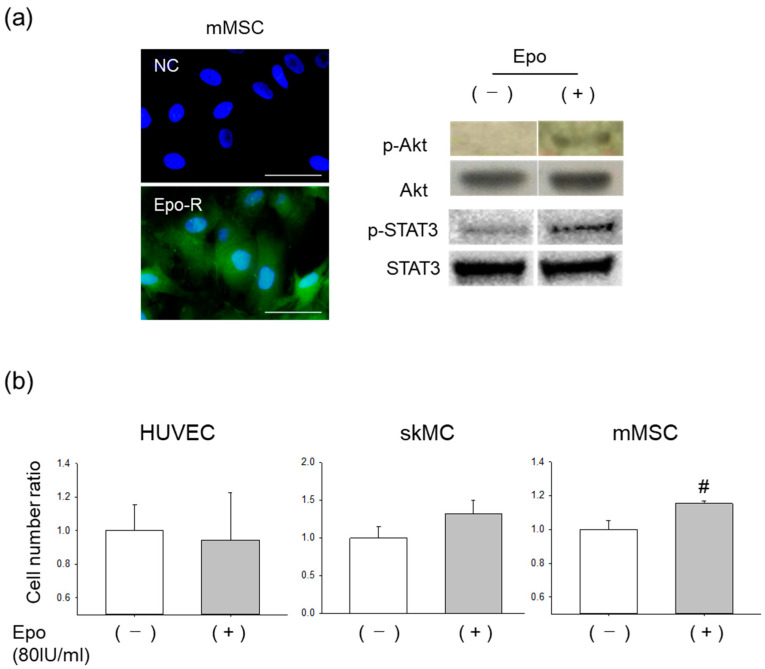
Proliferation of cultured mMSCs by Erythropoietin (Epo). (**a**) Left, Epo-R (green) expression in mMSCs. Nuclei were stained with DAPI (blue). NC, negative control. Bar = 50 µm. Right, western blot analysis. Epo stimulated the phosphorylation of Akt and STAT3 in the mMSCs. Epo-R, erythropoietin receptor. (**b**) Efficacy of Epo stimulation on the propagation of HUVECs, skMCs, and mMSCs. N = 3 to 6. # *p* < 0.01. HUVECs, human umbilical cord vein endothelial cells.

Epo-R was identified in the cultured mMSCs (Figure 3a). Recombinant Epo treatment was found to enhance the phosphorylation of Akt and STAT3 in the mMSCs. Thus, we tested Epo’s action on the proliferative activity of mMSC. HUVECs, skMCs, and mMSCs were treated with and without Epo in the appropriate media. Epo significantly increased the mMSC cell count when compared to that in the control levels, whereas propagation of the HUVECs was not induced (Figure 3b). The skMC cell count also tended to increase with Epo treatment, although the increase was not statistically significant.

### 3.3. PDGFR-α Positive MSCs in Rat Ischemic Limb Treated with Epo

**Figure 4:**
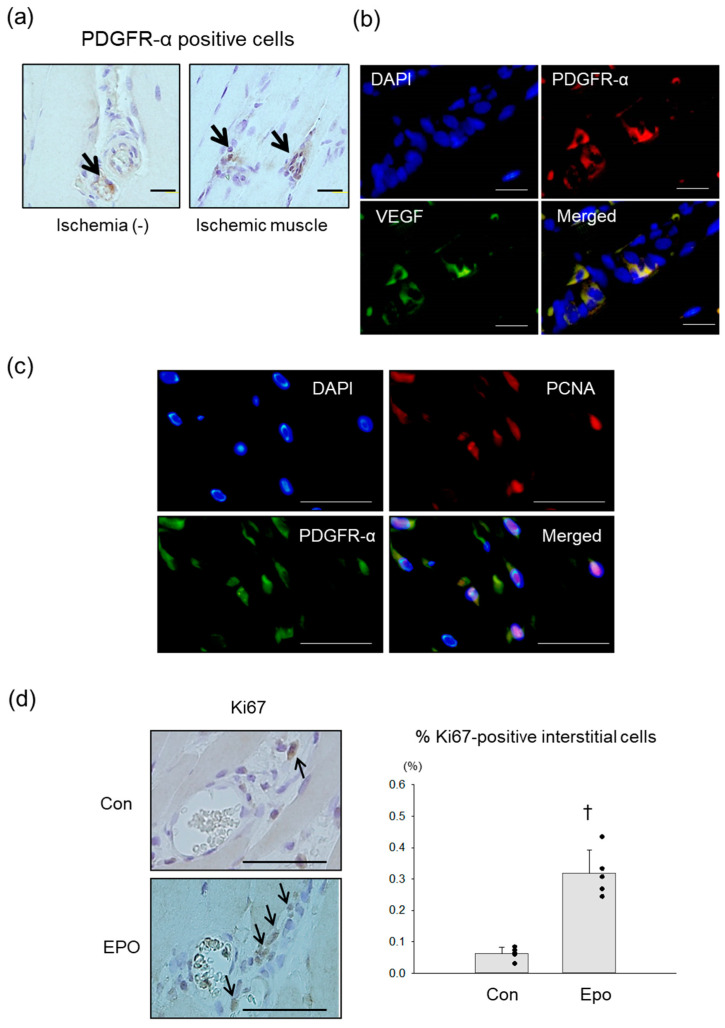
Proliferation of mMSCs in rat ischemic hindlimbs treated with Epo. (**a**) mMSCs positive for PDGFR-α (brown) were detected in the interstitial area of rat hindlimb muscles with and without ischemia. Arrows indicate the PDGFR-α-positive cells. Bar = 20 µm. (**b**) In double immunofluorescence, VEGF (green) was observed in mMSCs positive for PDGFR-α (red) in ischemic limbs. Nuclei were stained with DAPI (blue). Bar = 50 µm. (**c**) Proliferating cell marker, PCNA (red in the nucleus), was detected in the nuclei (DAPI, blue) of some of the mMSCs positive for PDGFR-α (green). Bar = 50 µm. PCNA, proliferating cell nuclear antigen. (**d**) Immunohistochemistry was performed with Ki67 antibody to evaluate the proliferating cells. Left images, the Ki67-positive cells (brown) in the nucleus were mainly detected in the stromal area around the vessels. Arrows indicate Ki67-positive cells. Bar = 200 µm. Right graph, comparison of the proliferating cell index between the ischemic limbs treated with and without Epo. N = 5. † *p* < 0.001.

PDGFR-α positive mMSCs were detected in the interstitial area of rat hindlimb muscles with and without ischemia, which was consistent with previous mouse and human studies [16,23] (Figure 4a). Double immunofluorescence revealed that the PDGFR-α positive mMSCs in the ischemic limbs expressed VEGF (Figure 4b).

We tested the in vivo effects of Epo by injecting Epo (5000 IU/kg) or vehicle directly into the ischemic limbs immediately and 7 days after the ischemic event since Epo directly induced the propagation of the mMSCs in our culture studies. A double immunofluorescence study performed to identify mMSC proliferation revealed the expression of PCNA, the proliferating cell marker, in the nuclei of the PDGFR-α positive cells in the stromal area (Figure 4c). Immunohistochemistry with Ki67 antibody was performed to quantify the proliferating cells. The Ki67-positive cells were detected in the stromal area around the vessels (Figure 4d). The proliferating cell index was defined as the ratio of Ki67-positive cells to the total number of cells in each perivascular interstitial area. On day 14, the index was significantly higher in the Epo-treated ischemic limbs than in the controls.

### 3.4. Angiogenic Effect of Epo in Rat Ischemic Limb

**Figure 5:**
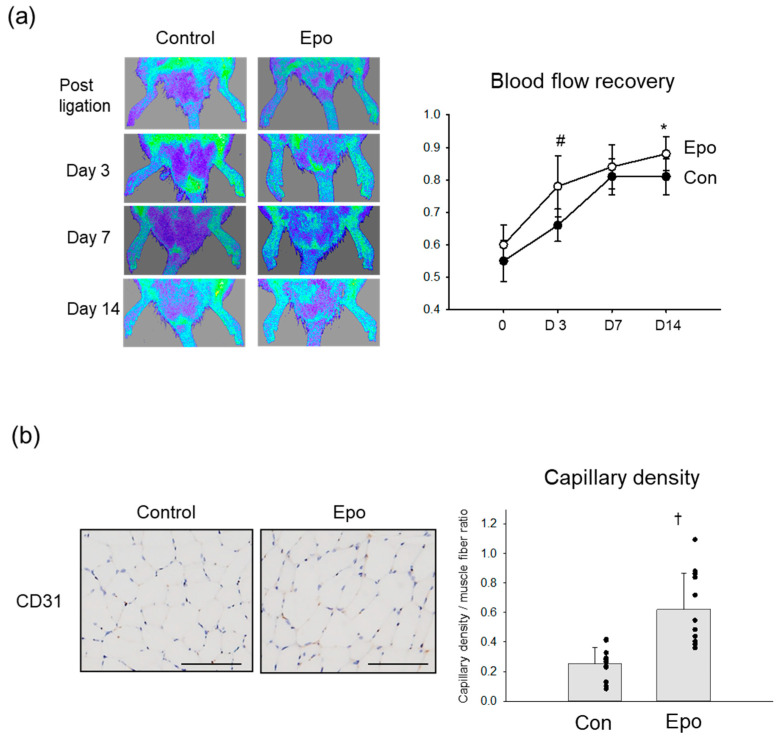
Angiogenic effect of Epo on rat limb ischemia. (**a**) Blood perfusion recovery assessed by LDPI. Left, representative images of LDPI. Right graph, time course changes of the LDPI indices in the Epo group and the controls. N = 11. * *p* < 0.05 vs. control on day 3. # *p* < 0.01 vs. control on day 14. (**b**) Left images, immunohistochemistry was performed using CD31 antibody to evaluate the capillaries in skeletal muscle on day 14. Right graph, comparison of the capillary density between the ischemic limbs treated with and without Epo. N = 11. † *p* < 0.001. Bar = 200 µm.

The angiogenic effect of Epo in ischemic rat limbs was also assessed by LDPI and histological studies. Blood perfusion recovery was more improved in the Epo groups than in the controls after the ligation (Figure 5a). In a quantitative analysis, the LDPI indices on days 3 and 14 after the ligation were significantly higher in the Epo group than in the control group. A histological study was performed to evaluate capillary growth in the muscle tissues on day 14 (Figure 5b). The capillary density assessed by CD31 staining was significantly higher in the ischemic muscle of the Epo-treated group than in the control group.

Taken together, the Epo treatment likely promoted neovascularization in the ischemic limbs in parallel with the induction of mMSC proliferation.

## 4. Discussion

Skeletal muscle has a remarkable regenerative ability after injury. Non-myogenic MSCs residing in skeletal muscle represent a distinct cell population from satellite cells and have been shown to play a vital role in muscle regeneration and homeostasis via interaction with satellite cells [16,17,18,23,24]. The**se** mMSCs are positive for PDGFR-α and are more frequently observed in the perimysium than in the endomysium, particularly in the perivascular space [24].

This study is the first to report the pro-angiogenic property of mMSCs and to describe the critical role that the mMSCs potentially play in promoting neovascularization after ischemic events in skeletal muscle. The mMSCs secreted VEGF and HGF, and the conditioned medium from the cells induced significant endothelial growth. Our previous studies have demonstrated the therapeutic efficacy of BMMSCs in animal models of cardiovascular disease via the paracrine action by secretion of repertoires of angiogenic factors [7,9,10,21]. VEGF was a crucial mediator of BMMSC-mediated effects in injured rats and porcine myocardium [9,25]. The levels of VEGF secreted from the mMSCs in this study were significantly higher than those secreted from the BMMSCs and skMCs.

Epo elicits not only a stimulating effect of erythropoiesis but also pleiotropic effects, such as mitotic and anti-apoptotic effects in various types of non-hematopoietic cells [26]. Epo has also been shown to mediate angiogenesis via various mechanisms [27]. However, controversy remains regarding whether Epo directly induces endothelial cell proliferation via Epo-R on the cells. Epo concentration**s** of 50 IU/mL reportedly stimulate endothelial cell proliferation, whereas higher doses do not [28]. An in vivo experiment reported that Epo promoted angiogenesis in the infarcted heart of mice, whereas an in vitro study to explore the underlying mechanisms demonstrated Epo treatment did not increase BrdU incorporation into HUVECs [29]. Propagation of endothelial cells induced by Epo was remarkably inhibited by anti-VEGF antibody [30], indicating an indirect effect of Epo on endothelial proliferation via VEGF production.

Our previous study demonstrated that Epo promoted BMMSC proliferation and survival under conditions of hypoxia and serum depletion [10]. Epo-R was also found in the mMSCs in this study as well as the BMMSCs. Epo treatment enhanced the phosphorylation of Akt and STAT3 in cultured mMSCs. While Epo induced the proliferation of cultured mMSCs in this study, it conferred no such effect on the endothelial cells. Epo promoted neovascularization in the ischemic rat limbs that we examined, and the mechanism behind the angiogenic effect appeared partly linked to the proliferation of mMSCs secreting angiogenic factors. Although other mechanisms of Epo-mediated angiogenesis, such as mobilization of endothelial progenitors from bone marrow [27], were not excluded, we surmise that the Epo-mMSC-VEGF axis could play a critical role in promoting angiogenesis in the Epo-direct injection models of this study. Exercise training has been shown to increase Epo expression in the myogenic cells of mice skeletal muscle [31]. One study described increased numbers of mesenchymal progenitor cells in the limb muscle of mice that were subjected to exercise [32]. One of the potential mechanisms underlying the benefits of exercise training in PAD is the induction of angiogenesis in skeletal muscle [33,34]. The benefits of exercise against muscle ischemia may therefore be conferred, at least in part, by the interaction between the myocytes and mMSCs via the Epo/Epo-R system.

These findings indicate the therapeutic potential of mMSCs in the activation of intrinsic reparative mechanisms in ischemic muscle. The activation of mMSCs by Epo injection directly into ischemic muscle tissue may become a novel strategy in angiogenic cell therapies. However, a previous study revealed that angiotensin-II and FGF-23 elicit premature senescence in mMSCs [19]. These neurohumoral factors are inversely associated with the pathophysiology of PAD and other diseases of the cardiovascular system [35,36]. Senescent MSCs could impair tissue homeostasis and repair, which may lead to imposing more deleterious effects. [37,38]. Adipose MSCs harvested from elderly with coronary artery disease exhibited reduced pro-angiogenic factor secretion [39]. Thus, the senescent phenotype of resident mMSCs may also contribute to the physiological decline of tissue homeostasis and regeneration due in part to the impaired pro-angiogenic property. To overcome this challenge, we surmise that strategies with the ex-vivo functional enhancement of the cells, such as Epo preconditioning, could become alternatives. Further experiments to address the efficacy will be necessary in the future since the primary focus of this study was the activation of the angiogenic system by stimulating mMSCs in ischemic limbs.

## 5. Conclusions

Taken together, muscle-derived MSCs exhibited a pro-angiogenic property and were activated by Epo in vitro and in vivo. mMSCs could therefore be an attractive therapeutic target, as their activation appears to be promising for PAD treatment. Although Epo could be one of the candidates involved in the activation of mMSCs, Epo also affects the hematopoietic system. Therefore, further studies are needed to explore or develop a specific mMSCs activator for a clinical application. 

## Data Availability

Not applicable.
